# Raising Money for Hospitals

**Published:** 1923-02

**Authors:** W. Fenn

**Affiliations:** Chaplain, Salisbury General Infirmary. General Infirmary, Salisbury


### Raising Money for Hospitals.

SIR,—Further to my letter which you so kindly published in your December issue, I should like to try and show how the scheme outlined in my letter would meet the condition described by Dr. Fleming in your January issue.

Consider the case of a small country town, which we will call Q, possessing a Cottage Hospital of its own, and situated upon the edge of the districts served by two large provincial hospitals (A and B), and sending patients to both. The financial secretaries of A and B will know how difficult it is to get adequate support from Q under such circumstances. If, however, Q had a branch of the Hospitals League, let us see as how the situation would be affected.

Let us, then, suppose that such an organisation exists at Q, that it possesses 2,000 members, and that its scale of payment is that in use in connection with the Radcliffe Infirmary, Oxford. I suggest that it is fair to regard each hunderd members as being made up as follows :—

| | | |
|---|---|---|
| 15 families averaging 5 in number and contributing 4d. per week ... | = | 75 |
| 15 individuals contributing 2d. per week ... ... ... ... ... | = | 15 |
| 10 individuals contributing 1d. per week ... ... ... ... ... | = | 10 |
| Total ... ... ... | = | 100 |

The weekly contributions received per hundred members would then be as follows :—

| | | | |
|---|---|---|---|
| 15 Family contributions of 4d. =4×15 pence ... ... ... | = | 5s. | 0d. |
| 15 Individual contributions of 2d. =15×2 pence ... ... ... | = | 2s. | 6d. |
| 10 Individual contributions of 1d. ... | = | | 10d. |
| Total ... ... ... ... ... | = | 8s. | 4d. |

The total revenue of the Branch would then be : Per week=8s. 4d.×20 ; total, £8 6s. 8d. Per year= £8 6s. 8d.×52 ; total, £433 6s. 8d. Subtract 20 per cent. to cover cost of collection and unavoidable losses, £86 13s. 4d. ; hence the nett revenue of Branch =£346 13s. 4d. I suggest that it is fair to suppose that not more than one in sixty of the members will upon the average require hospital treatment in any one year. And upon this basis the number of members of the Q Branch of the Hospitals League received into hospital annually would be thirty-three. And for the purpose of our illustration I suggest that they may be regarded as receiving treatment as follows :—

| | |
|---|---|
| At Q Cottage Hospital... ... ... ... | 16 |
| At Provincial Hospital A ... ... ... | 7 |
| At Provincial Hospital B ... ... ... | 5 |
| At a London Hospital, C, for special treatment ... ... ... ... ... ... | 2 |
| At other Provincial Hospitals, D, F and G, as the result of illness or accident while away from home ... ... ... ... | 3 |
| Total ... ... ... ... ... | 33 |

I suggest also that the cost of an average case at each class of hospital concerned may be regarded roughly as follows : At the great London hospitals, £16 ; at provincial or county hospitals, £12 ; at cottage hospitals, £8. Then, upon this basis, the cost of the treatment of the cases with which we are concerned would be as follows :—

| | | | |
|---|---|---|---|
| 16 cases at Q Cottage Hospital | 16× 8 | = | £128 |
| 7 „ Provincial Hospital A | 7×12 | = | 84 |
| 5 „ „ „ B | 5×12 | = | 60 |
| 2 „ London Hospital C | 2×16 | = | 32 |
| 1 case each at Provincial Hospitals D, F and G ... ... | 3×12 | = | 36 |
| Total ... ... ... | | = | £340 |

To meet this the Treasurer of the Q Branch of the Hospitals League has in hand £346 13s. 4d., which would be divided among the hospitals concerned in proportion to the sums they had spent upon the treatment of its members, each receiving a sum slightly greater than the total expended. This illustration is worked out only on the simplest lines, but I submit that it may be regarded as typical, and that, therefore, if a scheme working on lines similar to those suggested could be put into operation in every town, village and district, the problem of overlapping as well as that of hospital support would be in the way of being solved.

W. Fenn, Chaplain,
Salisbury General Infirmary.

General Infirmary,
Salisbury.

